# Oral Delivery of Astaxanthin via Carboxymethyl Chitosan-Modified Nanoparticles for Ulcerative Colitis Treatment

**DOI:** 10.3390/molecules29061291

**Published:** 2024-03-14

**Authors:** Wen Zhang, Xinping Zhang, Xinyi Lv, Ao Qu, Wenjing Liang, Limin Wang, Pei Zhao, Zijian Wu

**Affiliations:** 1 Tianjin Key Laboratory of Food Science and Biotechnology, School of Biotechnology and Food Science, Tianjin University of Commerce, Tianjin 300134, China; 2 Key Laboratory of Low Carbon Cold Chain for Agricultural Products, Ministry of Agriculture and Rural Affairs, Tianjin 300134, China

**Keywords:** astaxanthin, carboxymethyl chitosan, nanoparticle, anti-inflammatory, ulcerative colitis

## Abstract

The oral delivery strategy of natural anti-oxidant and anti-inflammatory agents has attracted great attention to improve the effectiveness of ulcerative colitis (UC) treatment. Herein, we developed a novel orally deliverable nanoparticle, carboxymethyl chitosan (CMC)-modified astaxanthin (AXT)-loaded nanoparticles (CMC-AXT-NPs), for UC treatment. The CMC-AXT-NPs were evaluated by appearance, morphology, particle size, ζ-potential, and encapsulation efficiency (EE). The results showed that CMC-AXT-NPs were nearly spherical in shape with a particle size of 34.5 nm and ζ-potential of −30.8 mV, and the EE of CMC-AXT-NPs was as high as 95.03%. The CMC-AXT-NPs exhibited preferable storage stability over time and well-controlled drug-release properties in simulated intestinal fluid. Additionally, in vitro studies revealed that CMC-AXT-NPs remarkably inhibited cytotoxicity induced by LPS and demonstrated superior antioxidant and anti-inflammatory abilities in Raw264.7 cells. Furthermore, CMC-AXT-NPs effectively alleviated clinical symptoms of colitis induced by dextran sulfate sodium salt (DSS), including maintaining body weight, inhibiting colon shortening, and reducing fecal bleeding. Importantly, CMC-AXT-NPs suppressed the expression of pro-inflammatory cytokines like TNF-α, IL-6, and IL-1β and ameliorated DSS-induced oxidative damage. Our results demonstrated the potential of CMC-modified nanoparticles as an oral delivery system and suggested these novel AXT nanoparticles could be a promising strategy for UC treatment.

## 1. Introduction

Inflammatory bowel disease (IBD) is a refractory gastrointestinal disorder characterized by chronic and recurrent inflammation. It encompasses two main forms, ulcerative colitis (UC) and Crohn’s disease (CD) [1]. IBD has significantly transformed over the years and has become a common ailment with a growing presence across the globe [2], leading to heavy burdens on public healthcare systems. Although the exact cause of UC is not yet fully understood, several factors are known to contribute to its development, including the activation of the mucosal immune system and consequent intestinal barrier function deficiency, gut microbial dysregulation, and pathological cytokine production [3]. Currently, there is no standard treatment for the disease; patients suffering from IBD are required to undergo lifelong medication therapy to manage their symptoms and prevent flare-ups [4]. However, the conventional options like small-molecule anti-inflammatory drugs and immunosuppressants, along with emerging biological therapies like biologics, antibiotics, and immunomodulators, fall short of providing a complete cure and are often hindered by their adverse effects [5]. The innovative therapeutic approach should be explored to solve the treatment bottleneck for low toxicity and efficient oral therapeutic drugs.

Astaxanthin (AXT), also known as 3,3′-dihydroxy-β,β′-carotene-4,4′-dione, is a kind of natural red lipophilic keto-carotenoid with excellent antioxidant, anti-inflammatory, and immunomodulatory activities [6,7]. Several preclinical studies have produced evidence to support the utilization of AXT as a therapeutic intervention for inflammatory diseases, including UC [8,9]. Nevertheless, AXT confronts some intractable problems in the oral process, such as poor gastrointestinal stability and water solubility, which limits its intervention effect on colitis [10]. In recent years, researchers have been focusing on encapsulating AXT within nanoparticles as a novel approach to enhance the bioavailability of AXT and thus, improve the effectiveness of nutrition interventions for UC. For example, Luo et al. demonstrated that astaxanthin-loaded nanoparticles have the ability to improve the bioaccessibility of astaxanthin and diminish the oxidative stress in a Caco-2 cell model [11]. Similarly, Li et al. developed astaxanthin-loaded polylactic acid–glycolic acid nanoparticles with a zeta potential of −9.8 mV and showed that the nanoparticles effectively decrease the levels of MDA, TNF-α, IL-1β, and IL-6, indicating a protective effect against acute colitis [12]. Additionally, a nano-powder formulation loaded with AXT, with a content of 2.9%, was also produced to achieve a more efficient antioxidant effect [13]. However, these liposomes and particulate systems are limited by low entrapment efficiency and loading capacity, poor stability, and weak drug-targeting capability.

Natural polymers, like chitosan, have attracted great attention in the field of site-specific drug delivery systems and enhancing the stability of nanoparticles [14]. Chitosan has been proven to possess outstanding properties including biocompatibility, biodegradability, adhesion, nontoxicity, and low immunogenicity [15]. Nonetheless, chitosan exhibits a disadvantage of limited solubility in water due to its rigid crystalline structure, which hinders its potential applications in various processes. To address the drawback, carboxymethylation is employed to prepare water-soluble derivatives of chitosan, enhancing its physical solubility and introducing an electric charge [14,16]. Carboxymethyl chitosan (CMC), a hydrophilic derivative of chitosan by carboxymethylation at either C-6 hydroxyl groups or/and the NH_2_ moiety, is recognized as an effective polymer for application in targeted drug delivery due to its advantages including solubility, absorbability, mucoadhesive property, negligible cytotoxicity, excellent biocompatibility, and sustained drug release [17,18,19]. The CMC microsphere-loaded hyaluronic acid/gelatin hydrogels had a high concentration of drug in the colon tissue for more than 24 h and inhibited the levels of pro-inflammatory cytokines in mice with colitis [20]. The CMC-CBA-doxorubicin conjugate functionalized nanoparticles were also produced for application in targeted drug delivery [21]. Additionally, the CMC hydrogels demonstrated exceptional pH-dependent drug release at pH values of 6.8 and 7.4, suggesting the hydrogels as suitable carriers for the colon-specific ornidazole drug [22]. Moreover, the surface modification with CMC leads to a substantial improvement in the stability of liposomes due to the formation of a tightly bound layer of water on the liposome’s surface resulting from the hydration of CMC [23,24]. In addition to the mentioned properties, the presence of numerous –COOH and –NH_2_ groups in CMC leads to its excellent solubility over a broad pH range, ensuring the stability of alpha-linolenic acid-loaded nanoliposomes even under different pH conditions and enabling sustained drug release capabilities [25]. Based on these advantages, CMC has promising potential as a “shell” material for nanoparticles in the field of oral drug delivery specifically for the treatment of colitis.

In this study, the CMC was coated on the surface of AXT-loaded nanoparticles, which endowed these nanoparticles with improved storage stability and sustained drug release properties. The CMC-modified AXT nanoparticles (CMC-AXT-NPs) exhibited superior antioxidation and anti-inflammatory abilities in vitro and could protect mice against dextran sulfate sodium (DSS)-induced colitis after oral administration. This study presented a novel approach to develop a food-related AXT delivery system for the oral treatment of colitis and provided a theoretical foundation for the use of CMC as an excellent coating material in the pharmaceutical and food industries.

## 2. Results and Discussion

### 2.1. Preparation and Characterization of CMC-AXT-NPs

**Figure 1:**
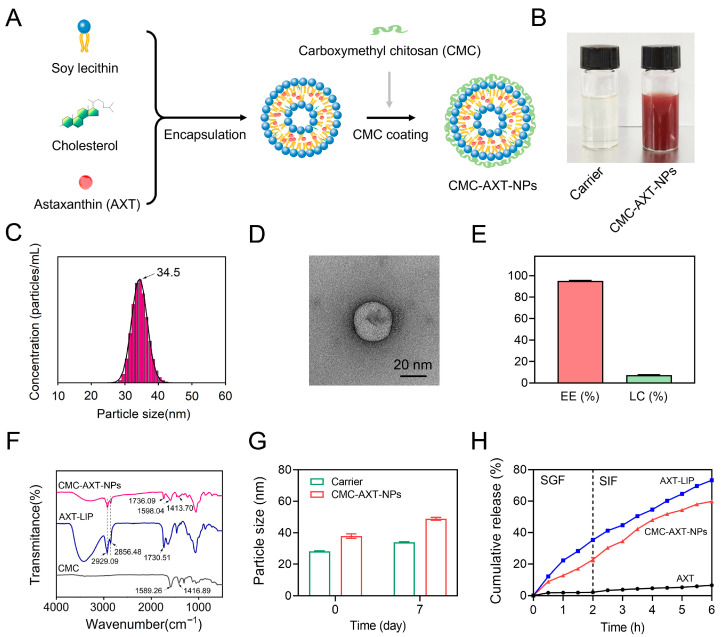
(**A**) Synthesis scheme of the CMC-AXT-NPs. (**B**) The appearance photograph of carrier and CMC-AXT-NPs. (**C**) Particle size distribution, (**D**) TEM images, (**E**) EE% and LC% of CMC-AXT-NPs. (**F**) FT-IR spectra of CMC, AXT-LIPs, and CMC-AXT-NPs. (**G**) Particle size of carrier and CMC-AXT-NPs after storage at 4 °C for 7 days. (**H**) In vitro release profile of AXT. Scale bar: 20 nm. Data are presented as mean ± SEM. AXT, astaxanthin; CMC, carboxymethyl chitosan; AXT-LIP, AXT-loaded liposomes; Carrier, CMC-modified AXT-unloaded nanoparticles; CMC-AXT-NPs, CMC-modified AXT-loaded nanoparticles; TEM, transmission electron microscopy; EE, encapsulation efficiency; LC, loading capacity; FT-IR, Fourier-transform infrared spectroscopy; SGF, stimulated gastric fluid; SIF, simulated intestinal fluid.

In a previous study, liposomes were designed as a delivery system for carotenoids in the form of oral supplements, which were found to enhance the bioaccessibility of carotenoids [26]. In order to improve the stability and sustained release capacity of the liposomes, carboxymethyl chitosan was coated on the surface of the AXT-loaded liposomes (AXT-LIP) (Figure 1A). The appearance photograph of CMC-modified AXT-unloaded nanoparticles (carrier) and CMC-AXT-NPs are represented in Figure 1B, and the result revealed that the freshly prepared CMC-AXT-NPs were uniform and stable, with no observable aggregation or agglomeration. In nanoparticle systems, the polydispersity index (PDI) is commonly used as a measurement to assess the distribution of particle diameters. A smaller PDI value signifies a narrower distribution of particle diameters, indicating a higher degree of uniformity in size among the particles [27]. In the case of the CMC-AXT-NPs, the PDI value was found to be 0.33 and the average size was estimated to be about 34.5 nm (Figure 1C). This low PDI value suggested that the particle diameter distribution of CMC-AXT-NPs was quite narrow, indicating a high level of uniformity in their size. Transmission electron microscopy (TEM) was then used to directly visualize the morphology of CMC-AXT-NPs, and the results were depicted in Figure 1D. Due to the coating of CMC, CMC-AXT-NPs appeared as nearly spherical shapes with rough, faint, and irregular edges (Figure 1D). The zeta potential of CMC-AXT-NPs was found to be approximately −30.8 mV, confirming the negatively charged nature of the nanoparticles. This negative charge, attributed to the presence of the CMC coating, played a crucial role in preventing aggregation of the nanoparticles through electrostatic repulsion, which is consistent with the findings previously reported [25]. Moreover, it was also found that a higher absolute value of negative charge is beneficial for the aggregation of nanoparticles in colon tissue [10].

Encapsulation efficiency (EE) is also a crucial parameter that determines the quality of nanoparticles [28]. After coating the nanoliposome with CMC, the EE of AXT reached a high value of 95.03%, and the loading capacity (LC) of AXT in the nanoparticles achieved a value of 7.31% (Figure 1E). This high encapsulation efficiency might be attributed to the fact that the CMC coating on the surface of the nanoparticles or its introduction within the phospholipid bilayer played a role in preventing the leakage of captured AXT, thereby improving the stability of AXT in the lipid phase [29]. Subsequently, the coating of CMC on the nanoparticles was investigated in detail using Fourier-transform infrared spectroscopy (FT-IR). As shown in Figure 1F, the absorption peaks at 1589.26 cm^−1^ and 1416.89 cm^−1^ of CMC were indicative of the bending vibration of –NH_2_ and –COO^–^ stretching vibration, respectively. After coating with CMC, the peaks of amino and carboxylic groups in CMC-AXT-NPs shifted to 1598.04 cm^−1^ and 1413.70 cm^−1^, respectively, which might be due to the hydrogen bonding interaction between CMC and bilayer [30]. Compared to AXT-LIP, the symmetrical stretching vibration absorption of C=O in CMC-AXT-NPs shifted from 1730.51 cm^−1^ to 1736.09 cm^−1^, which suggested hydrogen bonding between the carbonyl region of the lipid bilayer and CMC. Moreover, it was observed that the absorption peaks at 2929.09 cm^−1^ and 2856.48 cm^−1^, corresponding to antisymmetric and symmetric stretching vibration absorption of –CH_2_, respectively, did not change after coating with CMC, demonstrating that the interior structure of the bilayer remains unaffected by the presence of CMC [31]. The FT-IR results provided evidence that the CMC was successfully modified onto the nanoparticles.

Nanoliposomes have been found to be thermodynamically unstable, making them prone to degradation, aggregation, and fusion [32]. Therefore, achieving long-term stability is crucial for nanoparticles to be suitable for in vivo applications. To evaluate the stability of the CMC-AXT-NPs, the particle size of the nanoparticles was measured during storage at 4 °C. As presented in Figure 1G, the particle size of the CMC-AXT-NPs increased from 37.85 nm to 48.10 nm after 7 days of storage. This demonstrated that the CMC-modified nanoparticles exhibited improved storage stability, even when loaded with a high dose of AXT [33,34]. In addition, the in vitro simulated digestion of nanoparticles was also investigated using simulated gastric fluid (SGF) and simulated intestinal fluid (SIF). As depicted in Figure 1H, due to the extremely low aqueous solubility and dispersibility of AXT, the release of AXT was quite low, even in the simulated digestive fluids, which was consistent with the previous research [35]. By encapsulating AXT within nanoparticles, the release of AXT was significantly enhanced compared to its free form. The AXT-LIP exhibited a rapid release behavior during the first 2 h, with approximately 35.23% of the AXT released, which was probably caused by the swelling or disruption of AXT-LIP under gastric-acid conditions [36]. Furthermore, the negatively charged surface of AXT-LIP might facilitate the adsorption of positively charged ions in the surrounding medium, leading to the aggregation and destabilization of liposomes and further contributing to AXT release [37]. In contrast, the CMC-AXT-NPs displayed the slow-release behavior when exposed to gastric fluid for 2 h, with approximately 22.69% of the AXT released. This indicated that the CMC coating further enhanced the stability of nanoparticles under gastric conditions. The protective effect may be attributed to the presence of a large number of –COOH and –NH_2_ groups in CMC. The pH changes in the gastrointestinal tract influenced the conformation of the CMC molecular chain by affecting the amphiphilic groups, but most of these changes occurred in the outer layer of CMC, while the core of the nanoparticles remained shielded, ensuring the structural integrity of the nanoparticles [25]. With prolonged digestion time in simulated intestinal fluid, both AXT-LIPs and CMC-AXT-NPs exhibited a gradual increase in the cumulative amount of AXT released, reaching 73.21% and 59.80%, respectively, after 4 h of digestion. The results suggested that CMC-AXT-NPs were supposed to form a dense shell on the surface of nanoparticles, preserving their integrity and preventing the premature release of AXT [36]. As a result, a sustained and gradual release of AXT over an extended period of time could be achieved. Overall, these findings indicated that the CMC-AXT-NPs exhibit excellent encapsulation efficiency, long-term stability, and sustained release capacity in a simulated gastrointestinal environment. These properties make them highly suitable for potential application in oral delivery systems.

### 2.2. Cell Cytotoxicity Assessment

**Figure 2:**
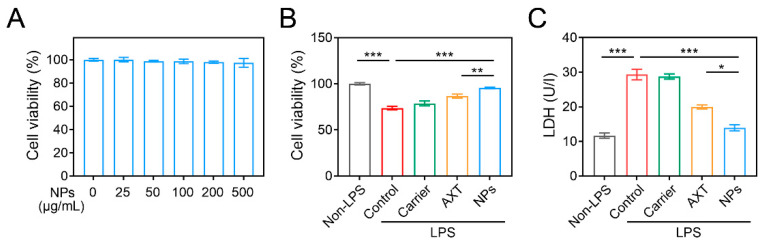
Cell cytotoxicity assessment. (**A**) Cell viability of Raw264.7 cells treated with different concentrations of CMC-AXT-NPs for 24 h. (**B**) Cell viability and (**C**) the level of LDH in Raw264.7 cells stimulated with LPS. Data are presented as mean ± standard error of the mean (SEM); * *p* < 0.05, ** *p* < 0.01, *** *p* < 0.001. NPs, CMC-AXT-NPs; LPS, lipopolysaccharide; LDH, lactate dehydrogenase.

As shown in Figure 2A, even at a higher concentration of CMC-AXT-NPs (500 μg/mL) for 24 h, there was no significant change in cell viability, suggesting that the nanoparticles had good biocompatibility and minimal side effects on Raw264.7 cells. To further investigate the potential benefits of CMC-AXT-NPs, we examined their effect on lipopolysaccharide (LPS)-induced cytotoxicity in Raw264.7 cells. As presented in Figure 2B, LPS induced significant cytotoxicity, with a cell viability of 73.6%. However, after incubating the cells with the carrier, AXT, or CMC-AXT-NPs, the cell viability increased to 78.6%, 86.6%, and 95.4%, respectively. This result clearly indicated that CMC-AXT-NPs had the ability to alleviate the cytotoxic effects induced by LPS in Raw264.7 cells. The lactate dehydrogenase (LDH) analysis, a widely accepted method to assess cell damage and membrane integrity, provided further support to these findings [38]. As depicted in Figure 2C, the level of LDH was significantly reduced in cells treated with CMC-AXT-NPs compared to those treated with carrier or free AXT. This suggested that CMC-AXT-NPs effectively protected the cells from the detrimental effects of LPS challenge. These findings demonstrated that CMC-AXT-NPs had the ability to effectively inhibit the LPS-induced cytotoxicity and promote the overall health and viability of cells.

### 2.3. In Vitro Antioxidant and Anti-Inflammatory Effects of CMC-AXT-NPs

**Figure 3:**
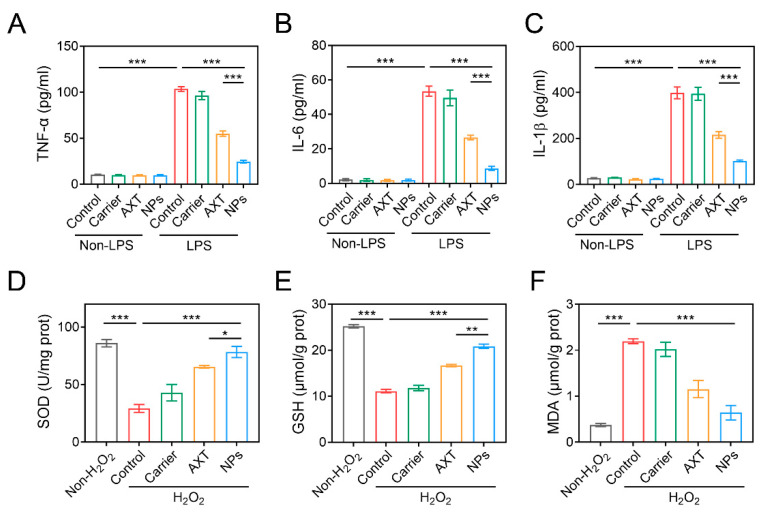
The inflammation factor levels of (**A**) TNF-α, (**B**) IL-6, and (**C**) IL-1β in Raw264.7 cells treated with LPS. The levels of (**D**) SOD, (**E**) GSH, and (**F**) MDA in Raw264.7 cells stimulated with H_2_O_2_. Data are presented as mean ± SEM; * *p* < 0.05, ** *p* < 0.01, *** *p* < 0.001. SOD, superoxide dismutase; GSH, glutathione; MDA, malondialdehyde.

Since oxidative stress and inflammation are particularly important contributors to the development and progression of IBD [39], we assessed the in vitro antioxidant and anti-inflammatory capacities of CMC-AXT-NPs by measuring the levels of H_2_O_2_-induced oxidative stress and LPS-induced inflammation in Raw264.7 cells, respectively. As shown in Figure 3A–C, the levels of TNF-α, IL-6, and IL-1β in the supernatant of cells stimulated with carrier, AXT, and CMC-AXT-NPs only were low and showed no significant difference compared to the non-LPS control group. This was consistent with the previous research, which demonstrated that AXT had limited anti-inflammatory effects under normal physiological conditions [40,41]. When stimulated with LPS, levels of TNF-α significantly increased compared to the non-LPS control group. However, both free AXT and CMC-AXT-NPs were able to correct this imbalance and reduce the levels of LPS-induced TNF-α expression. Notably, the cells treated with CMC-AXT-NPs showed significantly lower levels of TNF-α compared to those treated with free AXT, suggesting that the nanoparticles had a beneficial effect in relieving LPS-induced inflammation. Similar to TNF-α, the expression of IL-6 and IL-1β also significantly increased after LPS stimulation, with levels of 53.5 and 398.4 pg/mL, respectively. Fortunately, both free AXT and CMC-AXT-NPs were able to suppress the levels of IL-6 and IL-1β in LPS-treated Raw264.7 cells (Figure 3B,C), which might be due to the immunomodulatory effect of AXT under LPS stimulation [40,41]. It was important to note that the anti-inflammatory effect of AXT was dose-dependent, with high doses showing a better inhibitory effect on inflammation in LPS-stimulated cells [42]. The superior anti-inflammatory effect of CMC-AXT-NPs might be attributed to the fact that the NPs improved the release property and inhibited the degradation of AXT, thereby increasing the dosage of AXT in the cell culture supernatant. These findings demonstrated that CMC-modified AXT-loaded nanoparticles had excellent anti-inflammatory effects in Raw264.7 cells treated with LPS.

Oxidative stress refers to the imbalance between the generation of reactive species and the ability to counteract their harmful effects, which may lead to the disruption of biological systems [43]. When stimulated with H_2_O_2_, the levels of superoxide dismutase (SOD) and glutathione (GSH) in Raw264.7 cells significantly decreased compared to the non-H_2_O_2_ control group, indicating that the cells were unable to adequately neutralize the harmful effects of free radicals (Figure 3D,E). In contrast, the level of malondialdehyde (MDA) increased dramatically in the H_2_O_2_-control group (Figure 3F), suggesting an increase in lipid peroxidation and oxidative damage [44]. As expected, both AXT and CMC-AXT-NPs were effective in increasing the levels of SOD and GSH and decreasing the level of MDA. In agreement with the previous report, this protective effect was primarily attributed to the antioxidant properties of AXT [44,45]. Notably, the levels of SOD and GSH in the CMC-AXT-NP-treated group were approximately 1.2 and 1.25 times higher, respectively, than those in the AXT-treated group, indicating that the nanoparticles had a better effect in alleviating oxidative stress compared to free AXT (Figure 3D,E). Overall, these findings demonstrated that the CMC-AXT-NPs possessed superior antioxidation abilities and were effective in alleviating oxidative stress in vitro.

### 2.4. Anti-Colitis Activity of CMC-AXT-NPs

#### 2.4.1. Macroscopic Analysis of CMC-AXT-NPs on the Relief of Colitis

**Figure 4:**
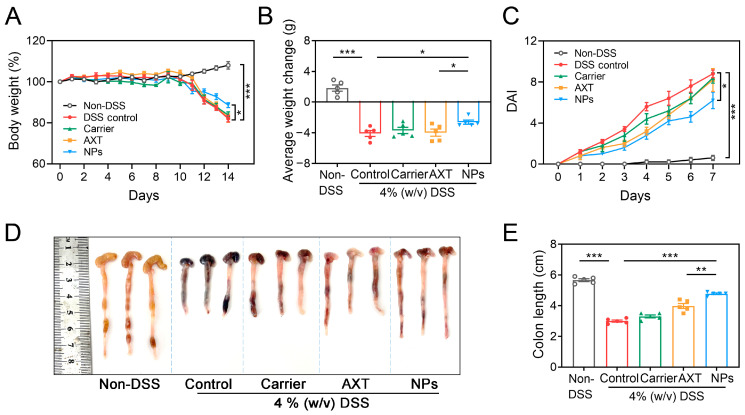
(**A**) Body weight, (**B**) average weight change, and (**C**) DAI in different treated groups. (**D**) Optical photographs of colons and (**E**) colon length in different treated groups. Data are presented as mean ± SEM; * *p* < 0.05, ** *p* < 0.01, *** *p* < 0.001. DSS, dextran sulfate sodium; DAI, disease activity index.

The therapeutic efficacy of CMC-AXT-NPs against acute colitis induced by 4% DSS was assessed by measuring body weights, colon lengths, and disease activity index (DAI) scores. As shown in Figure 4A and Table S1, it was observed that the body weight of mice in the DSS-control and carrier-, free AXT-, and CMC-AXT-NP-treated groups decreased by varying degrees from the third day of drinking water containing 4% DSS compared to that of the non-DSS control group. After 7 days of treatment, the body weight of mice in the DSS-control group decreased by 17.9% compared to the initial weight, which was consistent with the previous research [46]. Notably, the intervention of CMC-AXT-NPs resulted in a better preservation of body weight and reduced weight loss in mice (2.6 g) compared to free AXT (4.0 g), as shown in Figure 4B. These findings indicated that the AXT nanoparticles displayed enhanced capacity in maintaining body weight and reducing weight loss in colitis mice. Moreover, the DAI scores showed a time-dependent increase in DSS-control mice while exhibiting almost no change in the non-DSS control mice. However, mice treated with CMC-AXT-NPs exhibited significantly lower DAI scores compared to those treated with DSS alone, carrier, and free AXT, suggesting that CMC-AXT-NPs effectively alleviated colitis and reduced the inflammatory burden (Figure 4C, Table S2). It was observed that the colons of mice in the non-DSS control group appeared uniform and smooth without any signs of edema. In contrast, colons of mice in the DSS-control group exhibited edema, bleeding, and a significantly shortened length of 3.0 cm (Figure 4D). Interestingly, after oral administration of AXT and CMC-AXT-NPs, the symptoms of colonic shortening were reversed, and the length of the colon tissue increased to 3.9 cm and 4.7 cm, respectively (Figure 4E). These beneficial effects were mostly due to the potent anti-oxidant and anti-inflammatory activities of AST [47,48]. However, due to the poor water solubility and gastrointestinal stability of AXT, the potential biological activities were always unsatisfactory, even when taken in large doses through oral delivery [35,49,50]. In the CMC-AXT-NPs, AXT was protected from denaturation or degradation in the gastric acid environment and continuously released in intestine. Thus, a significantly improvement in the therapeutic effect strategy was achieved compared to free AXT. In summary, these results indicated that CMC-AXT-NPs effectively alleviated the clinical symptoms in mice with DSS-induced UC.

#### 2.4.2. Histological Observation of Colon Tissues

**Figure 5:**
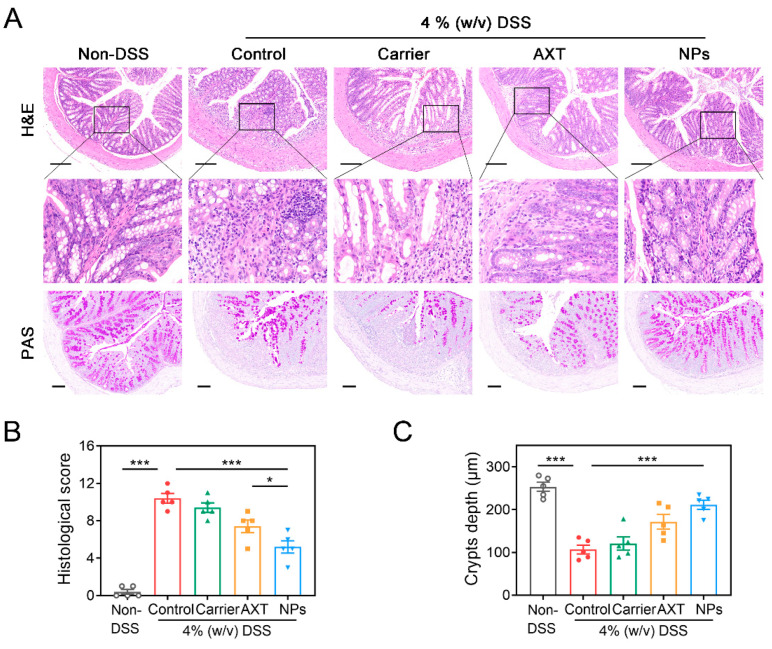
(**A**) H&E staining and PAS staining of colon tissues in different treated groups. (**B**) Histological score of colon tissues in different treated groups. (**C**) Crypt depth of colon tissues in different treated groups. Scale bar: 100 μm. Data are presented as mean ± SEM; * *p* < 0.05, *** *p* < 0.001. H&E, hematoxylin and eosin; PAS, periodic acid–Schiff.

As depicted in Figure 5A, the colon tissues in the DSS-control group exhibited extensive disruption of the crypt structure, along with prominent inflammation infiltration and ulceration compared to the non-DSS control group. However, the pathological damage of the colon tissues was significantly improved in the groups treated with AXT and CMC-AXT-NPs, mainly characterized by the presence of less-obvious ulcers, reduced inflammatory infiltration, and slight damage to the crypts. Interestingly, the group treated with CMC-AXT-NPs displayed almost normal histological structure with significantly reduced inflammation infiltration in the mucosa. Moreover, the histological scoring and analysis of crypt depth further supported the efficacy of CMC-AXT-NP treatment, as the mice treated with CMC-AXT-NPs had significantly lower scores and improved crypt depth compared to those treated with DSS alone (Figure 5B,C). This implied that AXT-loaded nanoparticles effectively intervened in the inflammation and crypt damage in the colon tissues. Furthermore, the colon tissues were stained with periodic acid–Schiff (PAS) to evaluate the thickness of the mucous epithelium and the presence of goblet cells. The groups treated with free AXT and CMC-AXT-NPs showed a mild improvement in the decrease in goblet cells, which further supported the results obtained from the histological observations and scores of the colon tissues (Figure 5B). In conclusion, the CMC-AXT-NPs demonstrated the ability to ameliorate the intestinal lesions induced by DSS treatment and exhibited superior capabilities in protecting and maintaining the integrity of the gastrointestinal tract compared to free AXT. These findings suggested the potential therapeutic application of CMC-AXT-NPs in the treatment of colon tissue inflammation and damage.

#### 2.4.3. Protective Effects of CMC-AXT-NPs on DSS-Induced Inflammation

**Figure 6:**
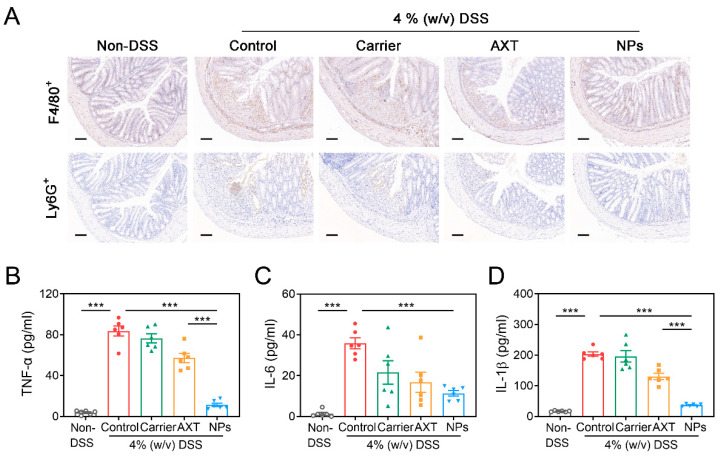
(**A**) Immunohistochemistry staining of F4/80 and Ly6G of colon tissues in different treated groups. The levels of (**B**) TNF-α, (**C**) IL-6, and (**D**) IL-1β in the serum of different treated groups. Scale bar: 100 μm. Data are presented as mean ± SEM; *** *p* < 0.001.

It is well-known that the immune dysfunction is the root cause of the dominating development of IBD [39]. In murine colitis models, there is an observed increase in the infiltration of monocytes and macrophages into the colon mucosa, which are responsible for the production of various inflammatory mediators [51,52]. In agreement with the previous report, there was a notable increase in the recruitment of neutrophils and macrophages in the colon tissues of the DSS-control group compared to the non-DSS control group (Figure 6A), indicating severe inflammation in the intestines [53]. Numerous reports revealed that by reducing the influx of inflammatory cells into the affected intestinal tissues, the overall inflammatory burden could be significantly decreased in DSS-induced colitis [53,54]. As shown in Figure 6A, intervention with AXT and CMC-AXT-NPs obviously alleviated the infiltration of neutrophils and macrophages in the colon tissues, suggesting that they had a good ability to maintain intestinal immune homeostasis and relieve intestinal tract injury [53,54]. There is accumulating evidence that pro-inflammatory cytokines, such as TNF-α and IL-6, play a crucial role in the development of IBD [55]. After administration of DSS, significantly higher levels of serum TNF-α, IL-6, and IL-1β were observed compared to the non-DSS control group, confirming the increasing trend of inflammation in the pathogenesis of colitis (Figure 6B–D). However, treatment with free AXT and AXT-loaded nanoparticles led to a decrease in the expression of pro-inflammatory cytokines compared to the mice in the DSS-control group, suggesting that AXT promoted the recovery of inflamed colon tissue by inhibiting the secretion of inflammatory cytokines (Figure 6B–D). Especially in the CMC-AXT-NP-treated group, the levels of TNF-α, IL-6, and IL-1β were reduced by 86.4%, 68.4%, and 81.9%, respectively, compared to those in the DSS-control group. The remarkably improved anti-inflammatory effects of CMC-AXT-NPs could be attributed to the preferable oral bioavailability and the sustained-release behavior of the nano-delivery systems [35]. In conclusion, these findings provided strong evidence that the anti-inflammatory activity of AXT was notably enhanced by employing CMC-modified nanoparticles as the delivery system.

#### 2.4.4. Antioxidant Effects of CMC-AXT-NPs

**Figure 7:**
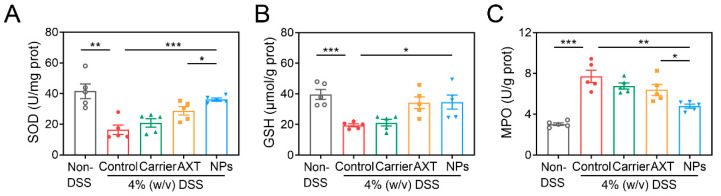
The (**A**) SOD activity, (**B**) GSH level, and (**C**) MPO activity in colon tissues of different treated groups. Data are presented as mean ± SEM. * *p* < 0.05, ** *p* < 0.01, *** *p* < 0.001. MPO, myeloperoxidase.

Numerous pieces of evidence suggest that IBD is linked to the increased production of reactive species [56]. Oxidative stress not only directly disrupts the intestinal barrier, which leads to cell dysfunction and tissue damage in the colon, but also causes dysregulation of multiple cell types, such as intestinal epithelial cells and immune cells [56,57]. To determine the impact of CMC-AXT-NPs on oxidative stress in mice with UC, the levels of oxidative stress markers were measured. The results in Figure 7A,B indicate that the levels of SOD and GSH significantly decreased after DSS administration compared to the non-DSS control group, indicating the occurrence of oxidative stress [58]. However, when AXT and CMC-AXT-NPs were supplemented, the levels of SOD and GSH significantly increased, demonstrating a potential antioxidant effect. It is worth noting that the level of SOD in the AXT-loaded nanoparticle-treated group was 1.3 times higher than that in the AXT-treated group. This improvement suggests that CMC-AXT-NPs were more effective in relieving oxidative stress compared to the use of free AXT. Various factors, such as enhanced stability, increased bioavailability, and controlled drug-release in the colon, may have contributed to this improvement. By utilizing CMC-modified nanoparticles, the efficacy of AXT was enhanced, resulting in a superior protective effect against DSS-induced oxidative stress. Moreover, treatment with CMC-AXT-NPs significantly reduced the levels of myeloperoxidase (MPO) compared to the DSS-control group and showed the most effective alleviation among all intervention groups (Figure 7C). In summary, CMC-modified AXT-loaded nanoparticles exhibited excellent antioxidant effectiveness in vivo, which was consistent with the findings in the cellular experiment.

## 3. Materials and Methods

### 3.1. Materials

Astaxanthin (purity > 90%), carboxymethyl chitosan (deacetylation degree > 85%), soybean lecithin, and cholesterol were purchased from Shanghai Yuan Ye Biotech Co., Ltd. (Shanghai, China). Lipopolysaccharide (LPS) and DSS (molecular weight 36,000–50,000) was obtained from Sigma-Aldrich (Shanghai, China). All other reagents used were of analytical grade.

### 3.2. Preparation of the CMC-AXT-NPs

The CMC-AXT-NPs were prepared using the thin-film hydration–high-pressure homogenization method [59]. Briefly, soybean lecithin, cholesterol, and astaxanthin (10:2:1, *w*/*w*) were dissolved in chloroform adequately and then evaporated on a rotary evaporator at 30 °C for 30 min to form a thin lipid film. Subsequently, the AXT-loaded nanoparticles were formed by hydrating the lipid film with phosphate buffer solution (PBS, 10 mL, 0.05 mol/L, pH 7.4) in a 55 °C water bath for 2 h. Next, carboxymethyl chitosan was dissolved in ultrapure water with a concentration of 1.5 mg/mL and injected into the same volume of AXT-loaded nanoparticles. The crude CMC-coated AXT-NPs were obtained by continuously stirring at 1000 rpm for 4 h. Finally, the CMC-AXT-NPs were achieved after high-speed shearing and high-pressure homogenization at a pressure of 150 Mpa for 5 cycles.

### 3.3. Characterization of CMC-AXT-NPs

The freshly prepared nanoparticles were properly diluted with ultrapure water before measurement. The particle size, PDI, and ζ-potential of CMC-AXT-NPs were determined using a Nanosight NS300 system (Malvern, UK) at 25 °C as previously reported (Pauluk et al. 2019) [60]. The morphology of CMC-AXT-NPs was checked by Talos F200C transmission electron microscopy (ThermoFisher, Waltham, MA, USA). Before observation, a drop of freshly prepared nanoparticle dispersion was added to the carbon-coated copper grids and dried at room temperature. TEM photographs were recorded at an accelerating voltage of 200 kV. The FT-IR spectra of samples were produced using an infrared spectrometer equipped with a DTGS detector and single reflection diamond ATR accessory (Bruker, Mannheim, Germany). For each spectrum, the samples were recorded at a resolution of 4 cm^−1^ from 500 to 4000 cm^−1^.

The encapsulation efficiency (EE) and loading capacity (LC) of nanoparticles was determined by centrifugal extraction (Tan et al., 2016) [59]. In brief, 1 mL CMC-AXT-NPs was mixed with 5 mL petroleum ether and stirred thoroughly for 5 min. The mixture was then subjected to centrifugation at 3000 rpm for 5 min. The organic phase (AXT extracts) was collected, and the absorbance at 490 nm was measured. The encapsulation efficiency and loading capacity were calculated by the following Equations (1) and (2), respectively:

(1) $$\mathrm{EE}\;(\%)=\frac{\mathrm{Encapsulated}\;\mathrm{AXT}\;\mathrm{content}}{\mathrm{Total}\;\mathrm{AXT}\;\mathrm{input}}\times 100$$

(2) $$\mathrm{LC}\;(\%)=\frac{\mathrm{Weight}\;\mathrm{of}\;\mathrm{AXT}\;\mathrm{in}\;\mathrm{NPs}}{\mathrm{Weight}\;\mathrm{of}\;\mathrm{NPs}}\times 100\;$$

### 3.4. Stability Analysis of CMC-AXT-NPs

To test the stability of CMC-AXT-NPs, the freshly prepared samples were sealed in brown glass bottles and stored at 4 °C for 7 days. The particle size of the nanoparticles was detected after storage.

### 3.5. In Vitro Release Profile of CMC-AXT-NPs

The in vitro release behavior of AXT from CMC-AXT-NPs was assayed as described previously with some modifications [60]. The sample solution (10 mL) was mixed with the same volume of stimulated gastric fluid (SGF) at 37 °C. Subsequently, the pH was adjusted to 2 and the mixture was stirred at 37 °C for 2 h. After that, the pH was adjusted to 7 and preheated simulated intestinal fluid (SIF) was added and reacted for another 4 h. The release rate of AXT in different digestion samples was measured at specified time points by UV/Vis spectroscopy at 490 nm. The cumulative release rate of AXT from CMC-AXT-NPs was calculated by Equation (3):

(3) $$\mathrm{Cumulative}\;\mathrm{release}\;(\%)=\frac{\mathrm{Total}\;\mathrm{amount}\;\mathrm{of}\;\mathrm{AXT}\;\mathrm{release}}{\mathrm{Total}\;\mathrm{amount}\;\mathrm{of}\;\mathrm{initial}\;\mathrm{AXT}\;\mathrm{in}\;\mathrm{NPs}}\times 100$$

### 3.6. Cell Cytotoxicity Assessment

Raw264.7 cells were seeded in 96-well plates (1 × 10^5^ cells per well) and cultured for 24 h. The carrier (250 μg/mL), AXT (125 μg/mL), and NPs (250 μg/mL) were added and incubated continuously for 24 h. LDH release was measured with a cytotoxicity assay kit (Promega, Madison, WI, USA, G1780). Cell viability was evaluated using a 3-(4,5-dimethylthiazol-2-yl)-2,5-diphenyltetrazolium bromide (MTT)-based colorimetric assay with a cell proliferation assay kit according to the manufacturer’s instructions (Promega, G3580) [61].

### 3.7. In Vitro Antioxidant and Anti-Inflammatory Evaluation

As for the antioxidant experiment, Raw264.7 cells were seeded in 6-well plates and the medium, comprising carrier (250 μg/mL), AXT (125 μg/mL), or NPs (250 μg/mL), was added and incubated for 12 h. Afterwards, the cells were stimulated with H_2_O_2_ (500 μmol/L) for another 12 h. Superoxide dismutase (SOD), glutathione (GSH), and malondialdehyde (MDA) were detected in accordance with the biochemical kit (Nanjing Jiancheng Bioengineering Institute, Nanjing, China). To detect the anti-inflammatory effect, Raw264.7 cells were seeded in 96-well plates and the medium containing carrier (250 μg/mL), AXT (125 μg/mL), or NPs (250 μg/mL) was added and incubated for 12 h. Subsequently, the cells were stimulated with LPS (1 μg/mL) and incubated for another 6 h. The content of TNF-α, IL-1β, and IL-6 in the culture supernatants were detected by ELISA kit (Lianke Biotech Co., Ltd., Hangzhou, China) according to the manufacturer’s instructions.

### 3.8. In Vivo Intervention of CMC-AXT-NPs on DSS-Induced Colitis in Mice

#### 3.8.1. Animals and Experimental Design

**Figure 8:**
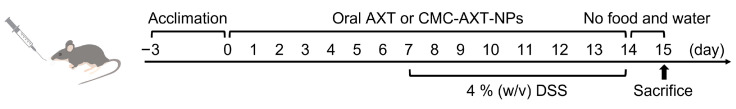
Schematic representation of UC experiment in vivo.

The potential therapeutic benefits of the oral administration of AXT nanoparticles for the treatment of UC were investigated in a mouse model induced by DSS [62]. The specific pathogen-free male C57BL/6 were obtained from Weitong Lihua Experimental Animal Technology Co., Ltd. (Beijing, China). Mice between 8 and 10 weeks were randomly allocated to different experimental groups: non-DSS control group, DSS-control group, carrier group, AXT group, and NP group. All experiments were conducted based on the schedule depicted in Figure 8. Briefly, after three days of acculturation, mice in the carrier, AXT, and NP groups were gavaged with carrier (20 g/kg/d), pure AXT, or NPs (dosage of AXT was 1 mg/kg/d), respectively, in the first weeks. Then, except the non-DSS control group, mice in the other groups were given freely a drink of 4% DSS for 7 days to induce colitis. The mice were anesthetized and sacrificed at the end point, and the serum and colons of the mice were harvested. All animal experiments were reviewed and approved by the Institutional Animal Care and Use Committee of the Chinese Academy of Medical Sciences and Peking Union Medical College.

#### 3.8.2. Evaluation of the Disease Activity Index (DAI)

The clinical progression of colitis in mice was assessed by the DAI score during the experiment. The DAI score was assessed by the scores of weight loss (0–4), fecal bleeding (0–4), and stool consistency status (0–4) according to the previous research [63].

#### 3.8.3. Histological and Immunohistochemical Assay

Colon tissues were excised and fixed in 4% paraformaldehyde and then embedded in paraffin. Sections were stained with hematoxylin and eosin (H&E) and periodic acid–Schiff (PAS). The histopathological scores were obtained by summating the four inflammatory parameters: inflammation severity, inflammation range, crypt damage degree, and the percentage of the area involved in inflammation, according to a previous study [62]. The immune cell infiltration in mice colon tissues was determined by immunohistochemistry staining with anti-mouse F4/80 or anti-mouse Ly6G antibodies. All images of the colon tissues were captured using Nikon Digital Sight DS-U3 camera (Nikon, Tokyo, Japan).

#### 3.8.4. Determination of Oxidative Stress and Inflammatory Factors

To detect the content of oxidative stress factors in the supernatant of colon tissues, SOD, GSH, MDA, and MPO biochemical kits were used according to the manufacturer’s instructions. The content of inflammatory cytokines (TNF-α, IL-1β, and IL-6) in the serum were detected using ELISA kits (Helsinki, Finland).

### 3.9. Statistical Analysis

All samples were conducted at least three times and the results are given as mean ± standard error of the mean (SEM). Statistical analysis was carried out using a standard two-tailed unpaired Student’s *t*-test with GraphPad Prism 7 (GraphPad Software, San Diego, CA, USA). *p* < 0.05 was considered statistically significant.

## 4. Conclusions

In the present study, novel orally deliverable CMC-modified nanoparticles were successfully developed for AXT delivery to alleviate the UC in mice. The CMC-AXT-NPs demonstrated exceptional functionality, with high encapsulation efficiency, preferable stability, and sustained drug-release properties. Furthermore, these nanoparticles exhibited excellent biocompatibility and were found to have minimal side effects, even at high concentrations. Additionally, CMC-AXT-NPs were able to effectively inhibit cytotoxicity induced by LPS in Raw264.7 cells. In vitro experiments confirmed that the CMC-AXT-NPs possessed superior antioxidant and anti-inflammatory abilities, suggesting that these nanoparticles had great potential for the treatment of IBD. Animal studies further substantiated these findings, as the CMC-AXT-NPs were able to effectively alleviate the clinical symptoms of colitis induced by DSS, including maintaining body weight, inhibiting colon shortening, reducing fecal bleeding, and promoting the repair of intestinal injury. Importantly, the oral administration of CMC-AXT-NPs effectively suppressed the secretion of pro-inflammatory cytokines, such as TNF-α, IL-6, and IL-1β, indicating that these nanoparticles had the ability to modulate the immune response associated with IBD. In addition, CMC-AXT-NPs showed remarkable antioxidant activity in vivo, increasing the levels of antioxidant enzymes such as SOD and GSH, while reducing the levels of colonic MPO. In conclusion, this study not only provided a promising strategy to enhance the therapeutic efficacy of AXT in treating UC, but also highlighted the potential of CMC-modified nanoparticles as a delivery system for other naturally derived antioxidants in the treatment of IBD.

## Data Availability

The raw data supporting the conclusions of this article will be made available by the authors on request.

## Supplementary Materials

The following supporting information can be downloaded at: https://www.mdpi.com/article/10.3390/molecules29061291/s1, Table S1: The percentage of body weight to the initial body weight of mice in different treated groups; Table S2: The disease activity index (DAI) of mice in different treated groups.
