# Supplementary material for: Oral Delivery of Astaxanthin via Carboxymethyl Chitosan-Modified Nanoparticles for Ulcerative Colitis Treatment
Source: Molecules. 2024 Mar 14;29(6):1291. doi: 10.3390/molecules29061291 (PMC10975021; doi:10.3390/molecules29061291)
Supplement: Supplementary file 1 [file molecules-29-01291-s001.zip › molecules-2900828-supplementary.pdf]

Supplementary Tables

Table S1. The percentage of body weight to the initial body weight of mice in different treated groups.

Days	Non-DSS (%)	4% (w/v) DSS			
		Control (%)	Carrier (%)	AXT (%)	NPs (%)
0	100	100	100	100	100
1	101.31±0.42	102.64±0.85	102.67±0.67	102.49±0.46	101.83±0.74
2	101.15±0.41	102.03±0.89	101.45±0.7	102.53±0.08	101.05±0.96
3	99.92±0.93	103±1.28	100.24±0.97	102.57±0.56	101.05±1.13
4	100.8±0.85	102.91±1.09	100.24±0.97	103.59±0.71	101.53±1.15
5	101.68±0.83	102.82±0.94	100.24±1.11	104.61±1.03	102.02±1.18
6	102.21±0.95	101.94±1.06	99.77±0.93	103.28±0.63	101.14±1.01
7	100.63±0.79	102.11±1.26	98.63±1.02	103.99±1.01	101.23±1.12
8	102.3±0.9	101.92±1.06	98.33±0.41	103.45±0.81	100.71±0.96
9	102.56±1.01	103.25±0.99	102.18±2.69	105.41±0.67	101.77±1.44
10	102.55±0.67	101.41±0.91	99.59±1.43	104.16±1.5	101.67±0.8
11	103.87±0.7	98.28±2.45	99.37±1.5	101.86±0.64	95.37±1.2
12	105.09±0.92***	90.73±1.68	91.39±2.14	92.44±1.3	95.02±1.46
13	106.67±1.06***	87.2±1.12	87.75±1.71	88.37±1.73	92.74±1.39*
14	107.99±1.77***	82.04±1.6	83.78±1.52	82.32±2.03	88.55±1.15*

Data are presented as mean ± SEM; Statistical analysis was carried out between DSS-control and different treated groups. *P < 0.05, ***P <0.001; DSS, dextran sulfate sodium; AXT, astaxanthin; Carrier, CMC-modified AXT-unloaded nanoparticles; NPs, CMC-modified AXT-loaded nanoparticles.

Table S2. The disease activity index (DAI) of mice in different treated groups.

Days	Non-DSS	4% (w/v) DSS			
		Control	Carrier	AXT	NPs
0	0	0	0	0	0
1	0***	1.2±0.2	1.2±0.2	0.8±0.37	0.8±0.2
2	0***	2.2±0.2	1.8±0.58	1.6±0.4	1.1±0.2**
3	0***	3.4±0.24	2.8±0.58	2±0.31*	1.6±0.24**
4	0.2±0.2***	5.6±0.4	4.4±0.51	3.2±0.37*	2.8±0.37**
5	0.2±0.2***	6.4±0.67	5.2±0.48	4.8±0.37	4.2±0.37*
6	0.4±0.24***	7.6±0.6	6.4±0.51	6.4±0.51	4.6±0.51**
7	0.6±0.24***	8.8±0.37	8.4±0.51	8.2±1.11	6.2±0.8*

Data are presented as mean ± SEM; Statistical analysis was carried out between DSS-control and different treated groups. *P < 0.05, **P < 0.01, ***P <0.001.
